# The awakening of dormant neuronal precursors in the adult and aged brain

**DOI:** 10.1111/acel.13974

**Published:** 2023-08-30

**Authors:** Bruno Benedetti, Maximilian Reisinger, Marie Hochwartner, Gabriele Gabriele, Dominika Jakubecova, Ariane Benedetti, Luca Bonfanti, Sebastien Couillard‐Despres

**Affiliations:** ^1^ Institute of Experimental Neuroregeneration, Spinal Cord Injury and Tissue Regeneration Center Salzburg (SCI‐TReCS) Paracelsus Medical University Salzburg Austria; ^2^ Austrian Cluster for Tissue Regeneration Vienna Austria; ^3^ Neuroscience Institute Cavalieri Ottolenghi (NICO) Orbassano Italy; ^4^ Department of Veterinary Sciences University of Turin Torino Italy

**Keywords:** action potential, aged brain, axon initial segment, dormant precursor, doublecortin, neurogenesis, neuronal precursor, synapse

## Abstract

Beyond the canonical neurogenic niches, there are dormant neuronal precursors in several regions of the adult mammalian brain. Dormant precursors maintain persisting post‐mitotic immaturity from birth to adulthood, followed by staggered awakening, in a process that is still largely unresolved. Strikingly, due to the slow rate of awakening, some precursors remain immature until old age, which led us to question whether their awakening and maturation are affected by aging. To this end, we studied the maturation of dormant precursors in transgenic mice (DCX‐CreER^T2^/flox‐EGFP) in which immature precursors were labelled permanently *in vivo* at different ages. We found that dormant precursors are capable of awakening at young age, becoming adult‐matured neurons (AM), as well as of awakening at old age, becoming late AM. Thus, protracted immaturity does not prevent late awakening and maturation. However, late AM diverged morphologically and functionally from AM. Moreover, AM were functionally most similar to neonatal‐matured neurons (NM). Conversely, late AM were endowed with high intrinsic excitability and high input resistance, and received a smaller amount of spontaneous synaptic input, implying their relative immaturity. Thus, late AM awakening still occurs at advanced age, but the maturation process is slow.

## Graphical abstract

Beyond the canonical neurogenic niches, the adult mammalian brain holds dormant neuronal precursors, which can remain immature until old age. In transgenic mice (DCX‐CreERT2/flox‐EGFP), we labelled immature dormant precursors at different ages, questioning whether awakening and maturation were taking place in the aged brain. We found that dormant precursors can awaken in old age and mature into fully functional neurons. Only slight differences distinguished early‐ and late‐matured neurons.

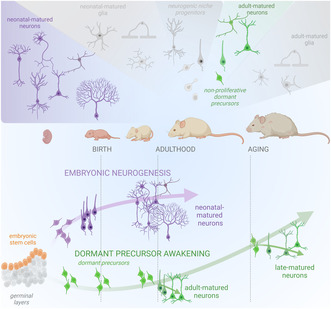

**Abbreviations**
AIS: axon initial segment
AM: adult matured neurons
AP: action potential
*C*_M_
: membrane capacitance
DCX: doublecortin
EGFP: enhanced green fluorescent protein
*E*_M_
: membrane potential
NM: neonatal matured neurons
PSC: postsynaptic current
*R*_in_
: input resistance
RMP: resting membrane potential
*R*_s_
: series resistance
Syn: synaptophysin
TAM: tamoxifen

## 1 INTRODUCTION

The mammalian brain is traditionally described as a network of neurons and glia, in which maturation and establishment of connectivity occur shortly after birth, followed by circuit refinement throughout the early part of life (Kast & Levitt, 2019). Accordingly, the apogee of brain maturation takes place sometime between puberty and early adulthood, followed by a progressive decline at the onset of aging. Yet, there are exceptions concerning the timing of maturation because specific types of neurons are added progressively to the brain circuits during the adulthood. Some well‐known neuronal “latecomers” are those originating from the brain areas designated as adult neurogenic niches (Brown et al., 2003; Couillard‐Despres et al., 2006; Ehninger & Kempermann, 2008; Emsley et al., 2005).

Recent studies and new technology allowed to update the current concepts of adult neurogenesis by revealing the existence of other types of neuronal precursors, which reside outside the neurogenic niches. Although generated during the embryonic development, these cell types retain post‐mitotic immaturity until adulthood (Benedetti & Couillard‐Despres, 2022; Bonfanti & Seki, 2021; Feliciano et al., 2015; La Rosa & Bonfanti, 2021). During adulthood, such neuronal precursors, herewith referred to as “dormant precursors,” eventually awaken and become adult‐matured neurons (AM). Much about the awakening and maturation of dormant precursors is yet to be revealed. So far, several works in different mammalian species suggested that dormant precursors occupy numerous brain regions (La Rosa et al., 2020; Luzzati et al., 2009; Piumatti et al., 2018; Sorrells et al., 2019). At the same time, tracing the fate and deciphering the role of these cells have been hindered by inherent limitations of labelling methods, which typically rely on histochemical markers of neuronal immaturity such as doublecortin (DCX) (Bonfanti & Nacher, 2012; Couillard‐Despres et al., 2005; Klempin et al., 2011; König et al., 2016; Marschallinger et al., 2014). Those limitations were overcome by the introduction of our murine model DCX‐CreER^T2^/flox‐EGFP mouse, allowing for a conditional recombination based on the DCX promoter (Couillard‐Despres et al., 2006; Zhang et al., 2010). In this model, immature dormant precursors can be labelled permanently and observed at time points past their immature state, thereby enabling the analysis of the process of dormant precursor awakening and maturation (Benedetti et al., 2019; Coviello et al., 2021; Rotheneichner et al., 2018).

We previously demonstrated that, after awakening, dormant precursors undergo axonal sprouting, formation of synapses, and the progressive acquisition of functional input and output during the transition from precursor to AM (Benedetti et al., 2019; Coviello et al., 2021; Rotheneichner et al., 2018). At the same time, we were puzzled to note that while many dormant precursors undertook the course of maturation during early adulthood, some cells remained immature throughout adulthood. Similar observation was also common in other mammalian species, including primates and humans (La Rosa et al., 2020; Sorrells et al., 2019). Consequently, we questioned whether the precursors remaining dormant for most of a lifetime can actually eventually awake and follow a course of late maturation or whether they fail to awaken altogether.

On the one hand, we speculated that the aging of the brain may hinder or completely impair the awakening. On the other hand, if late awakening and maturation were possible, this would imply that the old brain in mammalians is equipped with an unexplored source of young neurons.

## 2 MATERIALS AND METHODS

### 2.1 Transgenic animals

Transgenic DCX‐CreER^T2^/flox‐EGFP mice (Couillard‐Despres et al., 2006; Zhang et al., 2010) were used in order to label and track the fate of the dormant neuronal precursors in the piriform cortex. Through the administration of tamoxifen, this transgenic mouse strain allows for the permanent induction of the EGFP reporter gene expression in cells that express DCX. The tamoxifen administration (100 mg/kg of bodyweight, dissolved in corn oil, Sigma‐Aldrich) was carried out by oral gavage for five consecutive days. Experiments were performed in agreement with the “Directive 2010/63/EU of the European Parliament and of the Council of 22 September 2010 on the protection of animals used for scientific purposes” and were approved by Austrian animal care authorities: protocol number BMWFW‐66.019/0033‐WF/V/3b/2016.

### 2.2 Designation and definition of experimental groups

**FIGURE 1:**
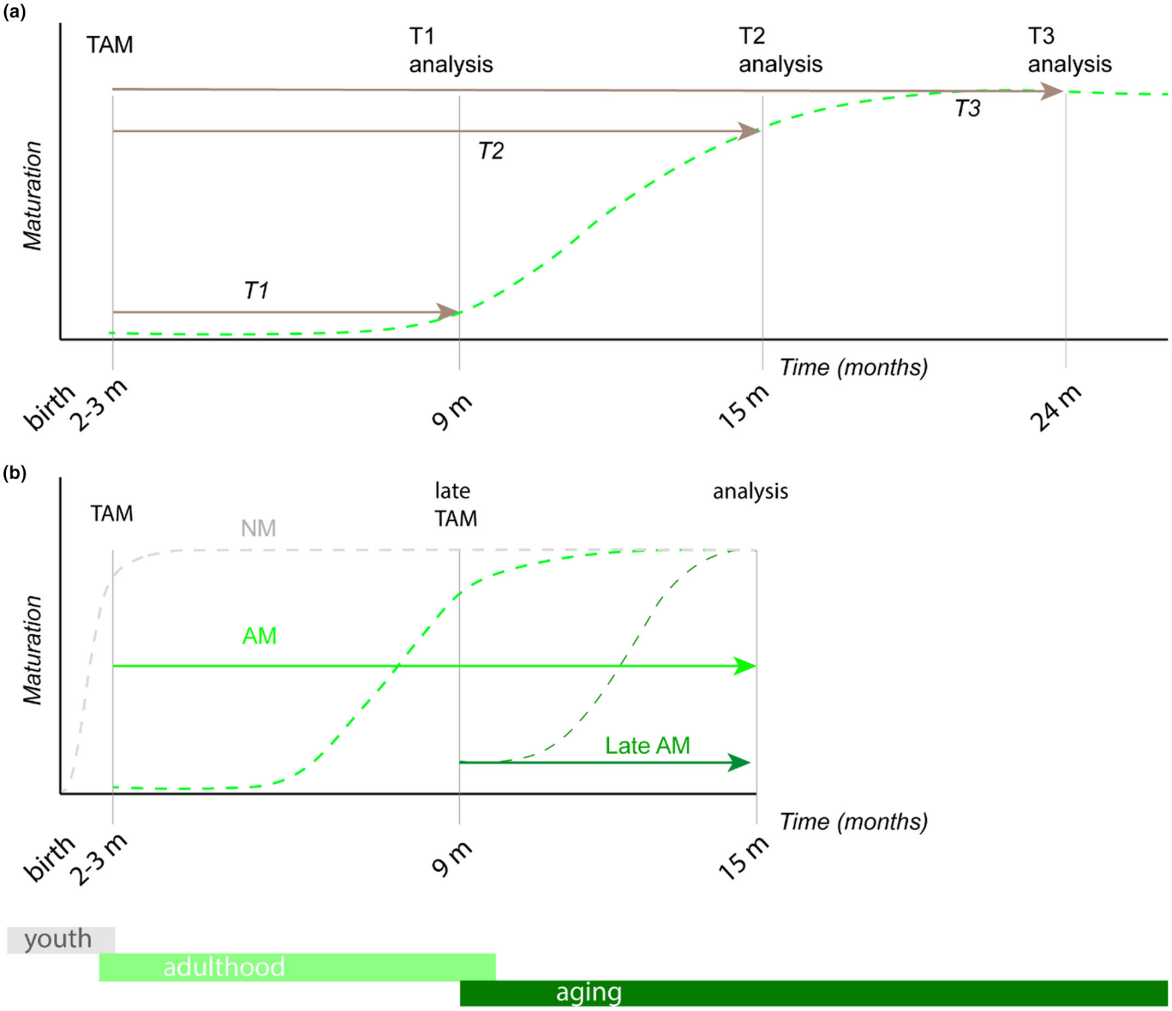
Designation and definition of experimental groups. (a) Graph highlights the designation of three experimental groups used to define age‐related effects on morphological traits of adult‐matured neurons (AM). Tamoxifen was administered at 2–3 months of age. Ex vivo analysis was carried out at 9 months (T1), 15 months (T2) and > 24 months (T3). (b) Graph highlights the designation of two experimental groups used to determine effects of age of maturation onset in relation to AM morphology and function. Tamoxifen was administered at 2–3 months of age (AM) or at 9 months of age (late AM). Analysis was carried out *ex vivo* at 15 months in both groups.

Questioning the time‐dependent features of dormant precursor maturation in relation to adulthood and aging (Figure 1a) TAM was administered at 2–3 months of age, and analysis was carried out in three cohorts (*N* = 5 mice/cohort) at 9 months (T1), 15 to 18 months (T2), and > 24 months (T3).

To analyze the morphological and functional traits of dormant precursors in relation to maturation onset, tamoxifen was administered at 2–3 months of age (AM) or at 9 months (late AM, Figure 1b). The age selection for late AM labelling was based on our previous knowledge that a residual fraction of immature AM persist until 9 months (König et al., 2016), whereas no DCX‐labelled cells were detected in the brain area of interest at 15 months. Analysis was carried out at 15 months in both AM and late AM. Traits of AM and late AM maturation were also compared with those of age‐matched neonatal‐matured neurons (NM).

### 2.3 Immunofluorescence and image analysis

Terminal (level 4) anesthesia was induced by intraperitoneal injection of ketamine (205 mg per kg bodyweight), xylazine (53.6 mg per kg bodyweight), and acepromazine (2.7 mg per kg bodyweight). Anesthetized mice were transcardially perfused with 0.9% NaCl for 5 min, followed by 0.1 M phosphate‐buffered 4% paraformaldehyde (pH 7.4) solution for 10 min. Brains were dissected and postfixed in paraformaldehyde solution for 2 h, followed by washout. Dissected brains were stored in 0.1 M phosphate buffer pH 7.4 at 4°C. Before sectioning, brains were transferred into 0.1 M phosphate‐buffered 30% sucrose solution (pH 7.4) at 4°C for at least 72 h. Brains were cut into coronal sections (40 μm) using a sliding microtome (Leica Microsystems) on dry ice. Until further processing, sections were stored at −20°C in cryoprotectant (25% v/v glycerol and sodium phosphate buffer at pH 7.4, 25% v/v ethylene glycol). Immunofluorescence detection was performed as described previously (Couillard‐Despres et al., 2006; Rubio et al., 2016). Primary antibodies used were chicken anti‐GFP (1:500, Invitrogen), rabbit anti‐DCX (1:300, Cell Signaling Technology), mouse anti‐PSA‐NCAM IgM (1:1000, Millipore), mouse anti‐NeuN (1:500, Millipore), guinea‐pig anti‐NeuN (1:500, Abcam), guinea‐pig anti‐Ank‐G (1:500, Synaptic Systems), rabbit anti‐β4‐spectrin (1:500, self‐made (Schlüter et al., 2017)), and mouse anti‐synaptophysin (1:500, Sigma‐ Aldrich). Secondary antibodies used were donkey anti‐chicken Alexa 488 (1:500, Jackson), donkey anti‐rabbit Alexa 568 (1:500, Invitrogen), goat anti‐mouse Alexa 647 (1:500, Molecular Probes), donkey anti‐mouse Alexa 568 (1:500, Invitrogen), and donkey anti‐guinea‐pig Alexa 647 (1:500, Jackson). Nuclei were counterstained with DAPI (Sigma‐Aldrich, 0.5 μg/mL). Fluorescence images were acquired using a LSM 710 confocal microscope and ZEN 2011 Basic Software (Carl Zeiss).

For image analysis, FIJI (Schindelin et al., 2012), an ImageJ‐based (National Institute of Health) platform, was used. The cell density of EGFP+ cells was calculated by manually counting the cells and dividing them by the volume of the respective imaged area in the tissue slice. The soma size, dendritic branching, and AIS length were measured by manual tracing. The dendritic spine density was calculated by determining the density of spines along non‐overlapping portions of EGFP+ dendrites at 50–1500 μm from the soma. The density of synaptophysin+ puncta juxtaposed to EGFP+ dendritic spines was calculated in relation to the total EGFP+ spine density along the same dendrites.

### 2.4 Electrophysiology

Before electrophysiological experiments, mice were anesthetized with isoflurane and decapitated. Brains were dissected while submerged in chilled artificial cerebrospinal fluid (ACSF). Coronal sections were sliced with a Leica VT1200s microtome at a thickness of 250 μm while submerged in chilled ACSF and transferred into a storage chamber, where they were submerged in room‐temperature ACSF. The setup for electrophysiological measurements consisted of an Olympus upright microscope. During experiments, brain slices were held in a chamber with a volume of 0.5–1.0 mL ACSF and an ACSF flow equal to 0.5–1.0 mL/min. ACSF used for slice storage and measurements of brain slices contained (in mM) 134 NaCl, 26 NaHCO_3_, 25.0 glucose, 2.0 CaCl_2_, 1.0 MgCl_2_, 2.4 KCl, and 1.25 NaH_2_PO_4_; pH was balanced to 7.4, using a mix of CO2/O_2_ (95/5%) (osmolarity = 315 mOsm). The chilled high‐sucrose ACSF used for slice preparation contained (in mM) 206.0 sucrose, 25.0 NaCO_3_, 25.0 glucose, 1.0 CaCl_2_, 3.0 MgCl_2_, 2.5 KCl, and 1.25 NaH_2_PO_4_. Osmolarity was equal to 309 mOsm (van Aerde & Feldmeyer, 2015). Patch pipettes had a resistance of 3.5–4.5 MΩ and allowed to achieve typical series resistance (Rs) of 20–30 MΩ. The intracellular pipette solution contained (in mM) 135 K‐gluconate, 4 KCl, 10 HEPES, 10 Na‐phosphocreatine, 4 ATP‐Mg, and 0.3 GTP‐Na (van Aerde & Feldmeyer, 2015). The pH was adjusted to 7.25 (osmolarity = 300 mOsm). Osmolarity was measured with a Vapro (Wescor) osmometer.

All experiments targeted cells of the posterior piriform cortex layer II (bregma −0.8 to −1.3). Recordings were acquired with a HEKA amplifier at 10 kHz, filtered at 2 kHz, and analyzed with FitMaster, Origin, PeakCount (courtesy of Dr Christian Hennenberger), and GraphPad Prism. Rheobase was determined with current clamp protocols, consisting of consecutive 500 ms‐long hyperpolarizing and depolarizing steps from resting membrane potential (RMP). Hyperpolarizing steps started at −20 pA, adding 5 pA to each consecutive step, until rheobase was reached. To determine the relationship between input current and action potential frequency, larger steps were used (20 pA), starting at −20 pA, and up to 250–300 pA. During inter‐step intervals, the membrane was kept at resting potential for 500 ms. During the inter‐step interval, the membrane was kept at resting potential for 500 ms. Series resistance (*R*_s_), input resistance (*R*_in_), and cell capacitance (*C*_M_) were derived from currents elicited by 50 ms voltage pulses as described in previous work (Benedetti et al., 2020). Postsynaptic current (PSC) frequency and amplitude were determined with voltage‐clamp experiments, using a holding potential of −70 mV. PSC detection was based on automatic thresholding criteria, using the software PeakCount, courtesy of Dr Christian Hennenberger (Rothe et al., 2018). The software uses the first derivative (slope) of PSC currents to automatically determine the local minimum in electric traces and the threshold of PSC detection is based on minimum slope amplitude. Based on empirically verified PSC detection accuracy, the detection limit was set arbitrarily to fivefold the standard deviation of the baseline. The accuracy of automatic threshold determination was further verified and found to detect events below an amplitude of 10 pA reliably.

### 2.5 Statistical comparisons

To test statistical significance in multiple comparisons, one‐way ANOVA analyses were followed by post‐test analyses and reported in graphs and tables as follows: *p < 0.05; **p < 0.01; ***p < 0.001. For comparisons between two sample groups, unless otherwise stated, unpaired *t* test was used (*p < 0.05; **p < 0.01; ***p < 0.001). Normality of sample distribution was assessed with the Kolmogorov–Smirnov test. Numerical data in the text and tables are reported as average ± standard deviation (SD). Box plots in figures represent median, interquartile distribution, and range.

For morphological characterization, confocal images were acquired from multiple regions of the piriform cortex, using five mice for each age group. In the tables documenting cell and spine density, N refers to the number of images analyzed. Spine density was quantified along a total dendritic length of 3000 μm to 3500 μM per age group. For the analysis of soma size, AIS length, and dendrites, N refers to the number of sampled cells, AIS, or dendrites, respectively.

## 3 RESULTS

### 3.1 Morphological AM maturation during adulthood and aging

**FIGURE 2:**
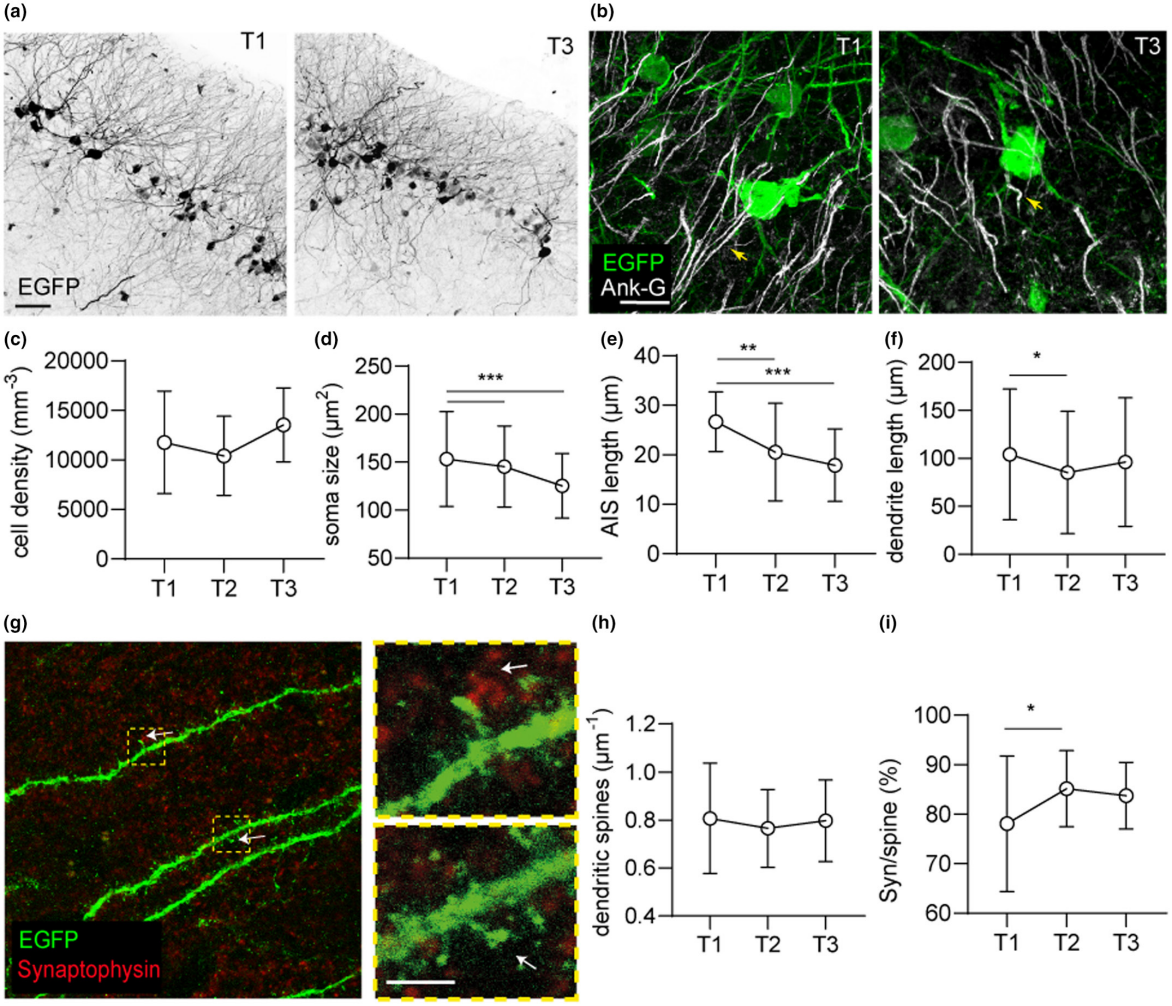
Morphological characteristics of adult‐matured neurons (AM) according to age. (a) The density, morphology, and neurite branching of AM was comparable between adult (T1) and aged brain (T3). (b) Subtle morphological differences were revealed by analysis of individual cells at different time points, for example, the axon initial segment (AIS; outlined by the detection of Ank‐G, highlighted by yellow arrows). (c) Density of labelled cells did not change significantly across ages. (d) Soma size of AM decreased significantly with age. (e) AIS length, as outlined by the scaffolding protein Ank‐G, decreased significantly with age in neurons expressing EGFP. (f) Dendrite length of labelled neurons decreased significantly between T1 and T2. (g) Juxtaposition of synaptophysin puncta (red) and EGFP+ synaptic spines on AM at different age allowed to estimate the degree of pre‐postsynaptic contacts. Top and bottom inset highlight a spine with and a spine without postsynaptic puncta respectively. (h) The density of dendritic spines did not change significantly with age. (i) The percentage of synaptophysin+ puncta juxtaposed to dendritic synapses increased with age, with significant difference detected between T1 and T2. *p < 0.05; **p < 0.01; ***p < 0.001.

We previously reported that dormant neuronal precursors of the posterior piriform cortex awaken during adulthood, mostly between the age of 3 and 9 months (Rotheneichner et al., 2018). While the onset of maturation is marked by the appearance of axons and synapses, concomitantly to the loss of immaturity marker expression (Benedetti et al., 2019), further maturation may consist in subtle and gradual processes of anatomical refinement. Therefore, we questioned which anatomical parameters outline the AM maturation during adulthood and aging. Permanent labelling of dormant precursors through the induction of EGFP expression (see Section 2) at approximately 3 months of age allowed to analyze the fate of these cells in the posterior piriform cortex layer II (Figure 2a,b) and compare their density and morphology, in mice of 9 months (T1), 15 months (T2), and > 24 months of age (T3).

The density of AM did not change significantly with age (Figure 2c; Table S1). However, significant age‐dependent changes in cell morphology were detected. Accordingly, we observed a significant shrinkage of the soma from T1 to T3 (Figure 2d; Table S1). Moreover, a significant shortening of the axon initial segment (AIS) between T1 and T3 was implied by analysis of Ank‐G distribution at the axon hillock of EGFP+ neurons (Figure 2b,e; Table S1). Furthermore, a slight, but significant shortening of dendritic branches occurred between T1 and T2 (Figure 2f; Table S1).

The density of synapses along EGFP+ dendrites was estimated by analyzing the relative abundance of synaptophysin+ puncta juxtaposed to synaptic spines (Figure 2g; Table S1). The density of synaptic spines on EGFP+ dendrites did not change significantly between T1, T2, and T3 (Figure 2h; Table S1). Conversely, the degree of synaptophysin+ puncta juxtaposed to EGFP+ spines increased with age, with a significant difference between T1 and T2 (Figure 2i; Table S1). Age‐related shrinkage of soma and shortening of the AIS also occurred in age‐matched NM (Table S2).

### 3.2 Morphological differences between early and late maturation onset

**FIGURE 3:**
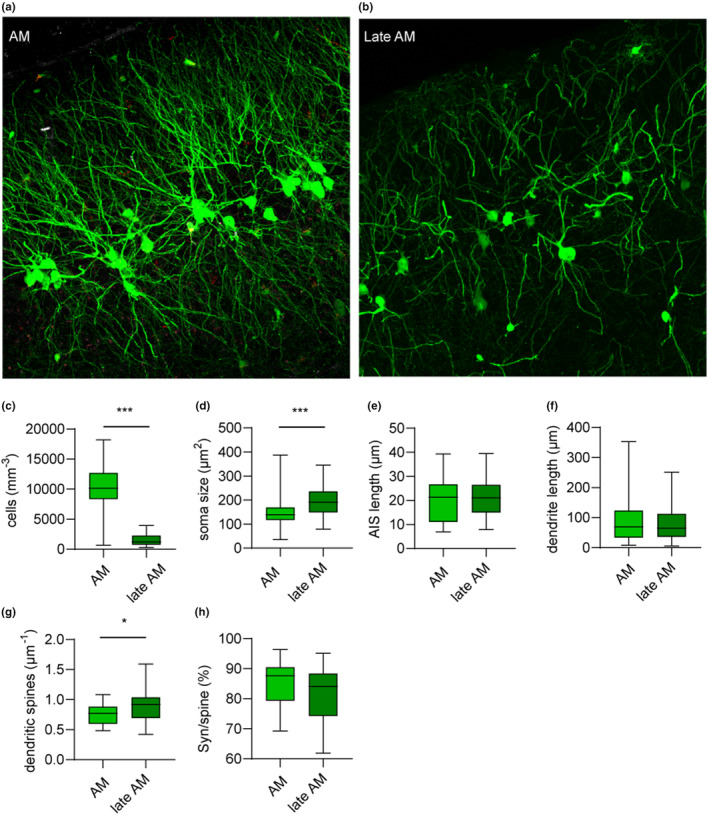
Morphological differences according to age of maturation onset. (a) Typical morphology of AM labelled at 3 months and analyzed at 15 months. Note the higher abundance of labelled cells in comparison to late AM in (b). (b) Typical morphology of AM with late onset of maturation, following labelling of dormant precursors at 9 months and analysis at 15 months. (c) Cell density of AM was significantly higher (by about 20‐fold) than that of late AM. (d) Soma size of AM was significantly smaller than that of late AM. (e) AIS length of AM and late AM were not significantly different. (f) Dendrite length of AM and late AM were not significantly different. (g) Density of dendritic spines in AM was significantly smaller than that of late AM. (h) The percentage of synaptophysin+ puncta juxtaposed to dendritic synapses in AM and late AM were not significantly different. *p < 0.05; **; ***p < 0.001.

On the one hand, the data presented so far imply a slow progressive maturation of dormant precursors throughout adulthood and aging. On the other hand, despite the fact that adult‐matured neurons (AM) awaken at an early age, some precursors maintain immaturity until old age, as revealed by previous work (Rotheneichner et al., 2018; Sorrells et al., 2019). Thus, we questioned whether the last immature fraction of late dormant precursors does eventually awaken and mature in the aged brain, becoming a new “late AM” cell population (see Section 2). We tackled this possibility by labelling the neuronal precursors that were still dormant at the age of 9 months, and we analyzed the morphology of late AM at 15 months. We readily observed that the EGFP‐labelled cells labelled in these conditions did mature (Figure 3 a, b), and developed large somata, abundant dendritic branching, AIS, and dendritic synapses. Moreover, both AM and late AM expressed NeuN, implying neuronal maturation (Data S1).

Analysis of cell density revealed that late AM represent a small fraction (ca. 14%) of the dormant precursors that were labelled at 3 months (Figure 3c, Table 1). Additionally, subtle morphological differences were detected between late AM and AM. Late AM were endowed with slightly larger soma compared with AM (Figure 3d, Table 1), whereas AIS length and dendritic length of AM and late AM were not significantly different (Figure 3e,f, Table 1). Late AM were also endowed with a slightly, but significantly higher density of dendritic spines than those observed in AM (Figure 3g, Table 1). Conversely, the proportions of synaptophysin+ puncta juxtaposed to synapses, as observed in AM and in late AM, were comparable (Figure 3h). Thus, even if aging did not hinder dormant precursor awakening and maturation, late maturation onset is associated with subtle morphological differences.

**TABLE 1:** Analysis of dormant precursor properties according to time of awakening.

	AM			Late AM			
	Average	SD	*N*	Average	SD	*N*	*p*‐values
Cell density (cell/mm^3^)	10.4*10^3^	4.0*10^3^	40	1.5*10^3^	0.9*10^3^	40	<0.001
Soma size (μm^2^)	145.4	42.24	1179	194.6	60.3	40	<0.001
AIS length (μm)	20.6	10.0	26	21.0	7.1	27	0.38
Dendrite length (μm)	85.2	63.7	187	82.33	60.4	259	0.35
Dendritic spines (spine/μm)	0.7	0.2	30	0.9	0.2	30	0.02
Syn/spine (%)	85.1	7.7	30	81.93	8.7	30	0.14

### 3.3 Increased intrinsic excitability results from late maturation

**FIGURE 4:**
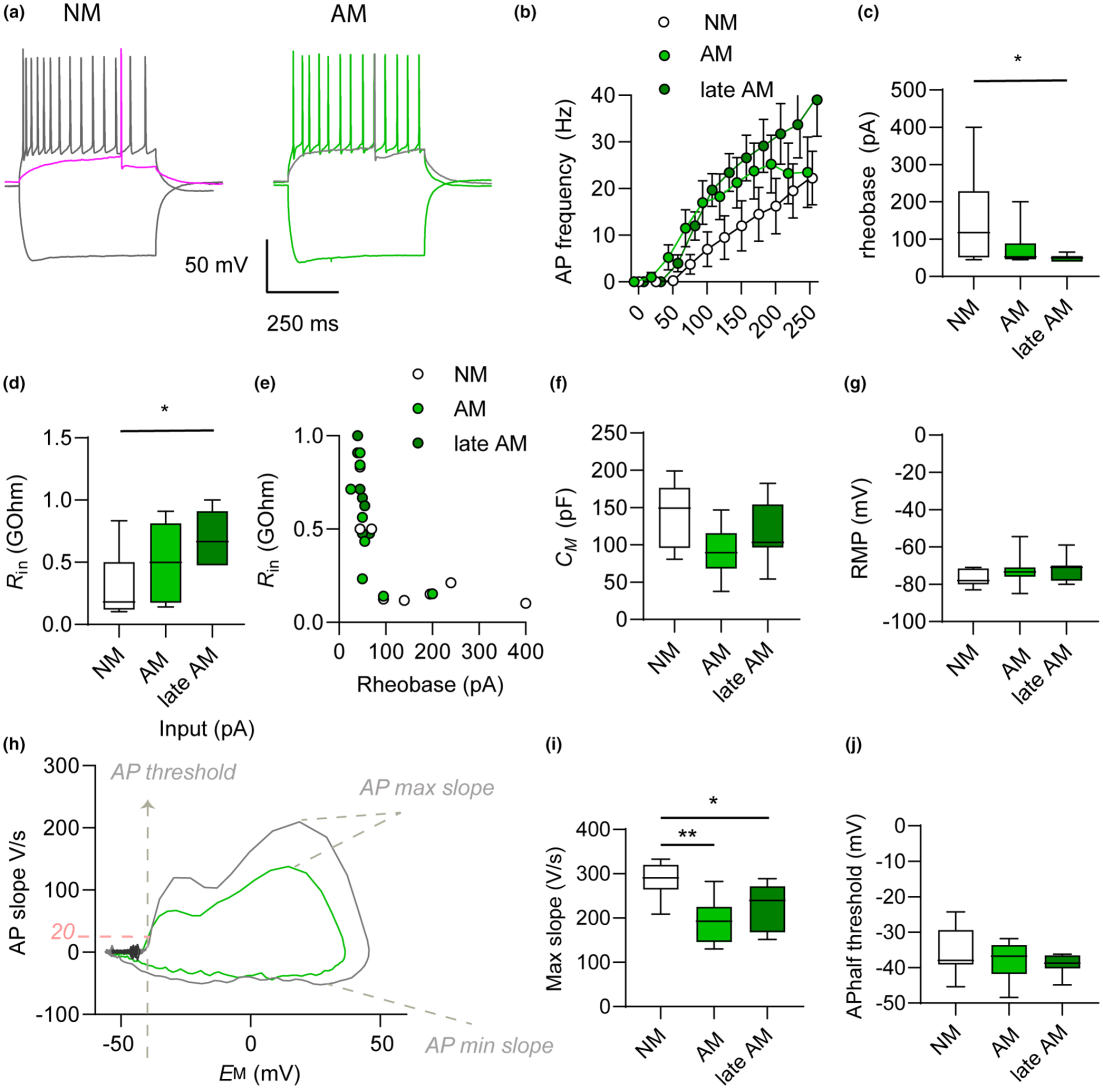
Action potential (AP) firing and intrinsic excitability of NM, AM, and late AM. (a) Typical patterns of action potential (AP) firing in NM and AM, evoked by chronic injection of current during single‐cell current clamp experiments. (b) Relation between the amplitude of input current injected and frequency of action potential firing showed the slightly higher intrinsic excitability of AM and late AM in comparison to NM; the differences were not significant. (c) The minimum input current necessary to elicit action potentials in NM was slightly larger compared with AM (not significant) and significantly larger compared with late AM. (d) The input resistance (*R*_in_) of NM was slightly smaller (not significant) compared with AM and significantly smaller than that of late AM. (e) The ohmic relation between *R*_in_ and rheobase is highlighted by individual data distribution showing coincidence of higher rheobase and lower *R*_in_. (f) The capacitance of NM was slightly larger (not significant) than that of AM and late AM. (g) The resting membrane potential (RMP) of NM, AM, and late AM were not significantly different. (h) Analysis of the first derivative of voltage over time (V/s) highlights the different kinetics (AP slope) in the AP of NM and AM. AP kinetics are displayed in relation to membrane voltage (*E*_M_). (i) The maximum slope of AP in NM was significantly higher than that of AM and significantly higher than that of late AM. (j) The AP thresholds of NM, AM, and late AM were not significantly different.

**FIGURE 5:**
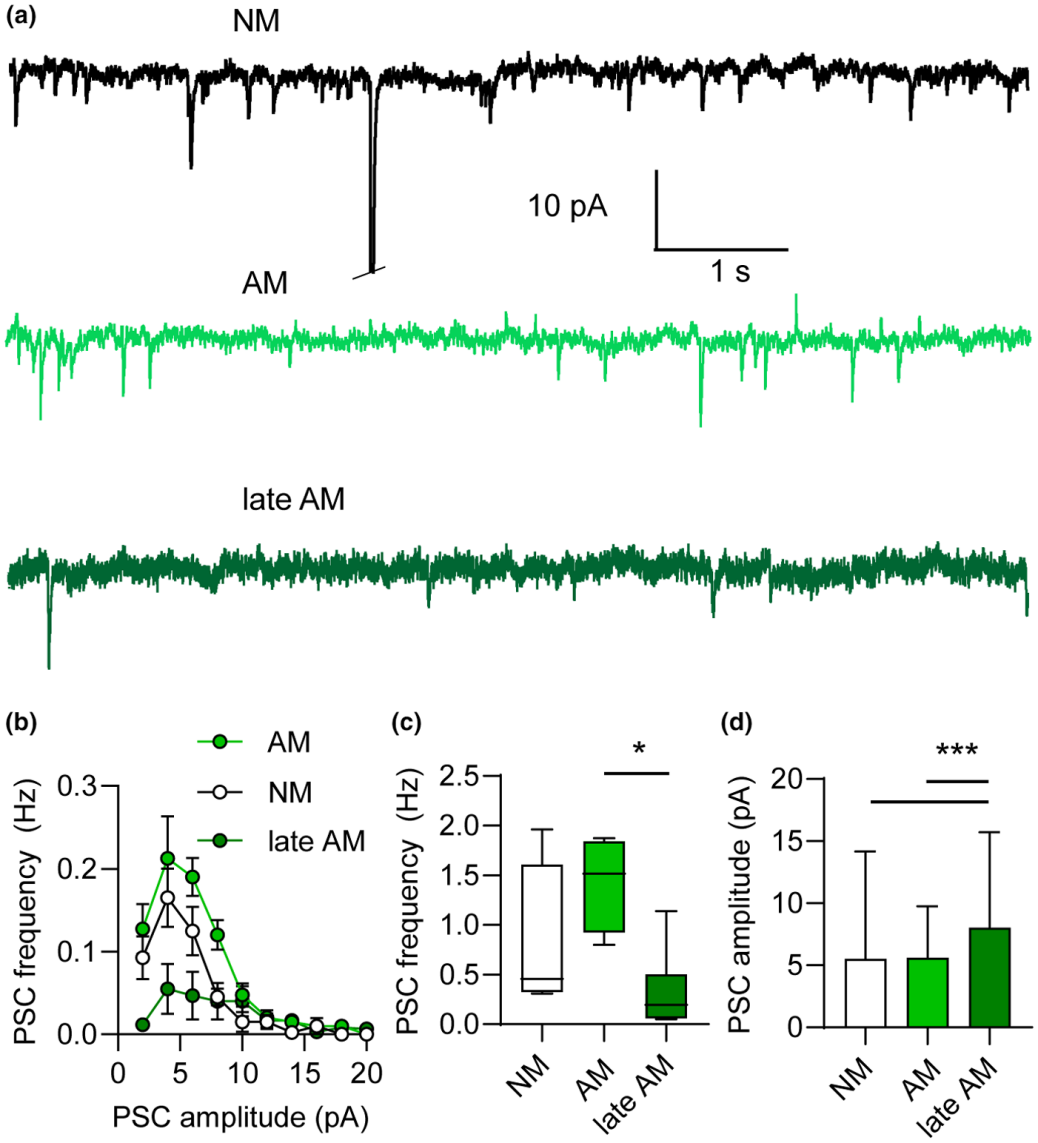
Spontaneous excitatory currents (PSC) received by NM, AM, and late AM measured near RMP (−70 mV) in single‐cell voltage‐clamp experiments. (a) Representative traces display spontaneous synaptic input in NM (black) and AM (light green) and late AM (dark green). (b) Histogram shows the distribution of PSC amplitude and frequency in NM, AM, and late AM. (c) The PSC frequencies of NM and AM are not significantly different. The PSC frequency of late AM is significantly lower than that of AM (d). The PSC amplitudes of NM and AM are not significantly different. However, the PSC amplitude of late AM is significantly larger than that of NM and AM.

On the one hand, our data imply that, following awakening, a subtle maturation process continues throughout life. On the other hand, some AM awaken late and experience a late maturation in the already‐aged brain. Therefore, we further questioned whether in virtue of different ages of awakening and different periods of maturation, the functional traits and degree of network integration of AM and late AM diverged. With single‐cell patch clamp experiments in acute brain slices, we explored physiological traits of AM, late AM and compared these cells to age‐matched neonatal‐matured neurons (NM). For all cell types, functional experiments were carried out at the age of 15 months (see Section 2). We found that AM, late AM, and age‐matched NM could readily fire action potentials (AP) upon chronic current injection (Figure 4a) and that all cell types received spontaneous synaptic input (Figure 5).

The relation between input (depolarizing current) and output (AP frequency) was largely comparable among NM, AM, and late AM (Figure 4b, Table 2). Notably, however, AM and late AM displayed a higher intrinsic excitability than NM, reflected by the smaller input sufficient to trigger AP firing (rheobase, Figure 4c, Table 2). The difference in intrinsic excitability was less pronounced and not significant comparing AM and NM (*p* = 0.146), but more prominent and significant comparing late AM and NM (*p* = 0.047). Among factors influencing the rheobase is the decrease in input resistance (*R*_in_) that usually accompanies neuronal maturation (Benedetti et al., 2020). Comparing NM to AM and late AM, we found that AM had a slightly higher *R*_in_ than NM (*p* = 0.482) and that late AM had a significantly higher *R*_in_ than NM (*p* = 0.045; Figure 4d, Table 2). The individual *R*_in_ distribution in relation to individual rheobase distribution was well fitted by a hyperbole, which recapitulates the ohmic relation between rheobase and *R*_in_ and suggests that *R*_in_ variability largely accounts for the different intrinsic excitability of the three cell types (Figure 4e, Table 2).

**TABLE 2:** Statistical analysis of dormant precursor and postnatal matured neurons: electrophysiological input/output properties and spontaneous synaptic input.

	NM			AM			Late AM			
	Average	SD	*N*	Average	SD	*N*	Average	SD	*N*	*p*‐values
Max AP frequency (Hz)	34.5	9.1	8	34.25	18.6	8	41.4	21.7	7	0.9769^a^ 0.8101^b^ 0.8101^c^
Rheobase (pA)	153.8	121.9	8	76.2	52.8	8	49.3	8.9	7	0.1461^a^ 0.7881^b^ 0.0468^c^
*R*_in_ (GΩ)	0.32	0.26	8	0.50	0.30	8	0.69	0.20	7	0.4827^a^ 0.8410^b^ 0.0448^c^
*C*_M_ (pF)	140.0	42.9	8	91.6	33.4	8	116.5	41.8	7	0.0661^a^ 0.4202^b^ 0.4202^c^
RMP (mV)	−76.7	4.5	8	−72.0	9.1	8	72.0	6.9	7	0.4958^a^ 0.9931^b^ 0.4958^c^
Max slope (V/s)	286.2	41.6		193.9	50.0	8	224.4	50.9	7	0.0028^a^ 0.2296^b^ 0.0409^c^
Min slope (V/s)	−57.6	8.6	8	−51.2	11.5	8	−54.9	12.6	7	0.5930^a^ 0.7759^b^ 0.7759^c^
AP half‐width (ms)	1.58	0.16	8	1.56	0.28	8	1.36	0.23	7	0.8294^a^ 0.2040^b^ 0.2053^c^
AP threshold (mV)	−35.8	6.9	8	−37.9	5.5	8	−39.2	2.9	7	0.6936^a^ 0.6936^b^ 0.5631^c^
PSC frequency (Hz)	0.8	0.7	4	1.4	0.5	4	0.3	0.4	6	>0.9999^a^ 0.0261^b^ 0.26689^c^
PSC amplitude (pA)	5.5	8.6	328	5.6	4.1	624	8.0	7.7	198	0.8390 <0.0001 <0.0001

^a^ Post hoc test comparison of NM vs. AM.

^b^ Post hoc test comparison of AM vs. late AM.

^c^ Post hoc test comparison of NM vs. Late AM.

Differences in *R*_in_ and intrinsic excitability might reflect different capacitance (*C*_M_) in relation to different cell size, and/or might reflect differences in resting membrane potential (RMP). However, NM had only a slightly larger *C*_M_ compared with AM (*p* = 0.066) and to late AM (*p* = 0.420; Figure 4f, Table 2). Additionally, the RMP was comparable among NM and AM (*p* = 0.496) and among NM and late AM (*p* = 0.496; Figure 4g, Table 2). Thus, in this case, neither changes in *C*_M_ nor in RMP could justify the different *R*_in_ and intrinsic excitability among cell types.

A further difference between the three cell types emerged when comparing AP kinetics (Figure 4h). NM were endowed with significantly quicker AP upstroke compared with AM and late AM (Max slope, Figure 4i, Table 2). However, no differences occurred in relation to other kinetic parameters, including AP threshold (Figure 4j, Table 2), half‐width, and minimum AP slope (Table 2).

### 3.4 Smaller functional synaptic input associated with late maturation onset

Since the AM and late AM were endowed with numerous dendritic synapses juxtaposed to synaptophysin+ puncta, we questioned the extent of functional network integration and compared the spontaneous synaptic input received by AM and late AM, to the input received by age‐matched NM (Figure 5a). Analysis of spontaneous postsynaptic currents (PSC) revealed that the excitatory input received by AM and NM were comparable (Figure 5b–d,) both in terms of amplitude (Figure 5c, Table 2) and frequency (Figure 5d, Table 2). Conversely, the input received by late AM was sparse, with a PSC frequency equal to less than half of the PSC frequency measured in the two other cell types. Thus, the frequency of synaptic input in late AM was significantly lower than that of AM (Figure 5c). At the same time, the PSC amplitude in late AM was significantly larger than the PSC amplitude in both AM and NM (Figure 5d).

## 4 DISCUSSION

The canonical neurogenic niches (hippocampus and sub‐ventricular zone) provide new neurons to the adult mammalian brain and have been extensively studied. In these regions, neurogenesis relies on a proliferative pool of neural stem cells, which is self‐regenerating (Bonaguidi et al., 2011; Doetsch et al., 1999). Although the role of neurogenic‐niche progenitors has been extensively explored, it is still the source of lively debates (Bonfanti & Amrein, 2018; Sorrells et al., 2021). In addition, the mammalian brain is equipped with dormant neuronal precursors able to undergo maturation during the whole lifespan of the individual (Benedetti & Couillard‐Despres, 2022; Feliciano et al., 2015). In contrast to the proliferative neurogenic‐niche progenitors, dormant neuronal precursors are an exhaustible pool of post‐mitotic cells scattered across several brain areas, with species‐specific abundance and patterns of distribution (König et al., 2016; La Rosa et al., 2020). The existence of non‐proliferative precursors outside the neurogenic niches has drawn increasing awareness only recently, especially after reports revealed that these cells can awaken and become mature neurons in the adult brain (Benedetti et al., 2019; Benedetti & Couillard‐Despres, 2022; Bonfanti & Seki, 2021; Coviello et al., 2021; Rotheneichner et al., 2018). Much is still to be deciphered in regard to their physiological role(s). Yet, our current work showed that most of the dormant precursors awaken and mature into neurons, some of them up to advanced ages.

A striking peculiarity of some dormant precursors is their capacity of retaining immaturity for most of the individual's lifespan, resulting in years or even decades of quiescence according to species longevity (La Rosa et al., 2020; Rotheneichner et al., 2018; Sorrells et al., 2019). As a consequence, while many awakening events occur during youth, some non‐proliferative precursors remain immature until the old age (Rotheneichner et al., 2018; Sorrells et al., 2019). In our work, we now showed that, regardless of old age and extremely protracted immaturity, even the late precursors do ultimately awake and mature into bona fide neurons, with only small differences teasing apart AM, late AM, and NM. Growing cell soma and AIS sprouting/elongation are morphological features generally associated with neuronal maturation as we reported earlier in relation to the transition from dormant precursor to neuron. Such changes usually occur in weeks for dormant precursors and NM alike (Benedetti et al., 2016, 2019, 2020; Coviello et al., 2021; Ghibaudi et al., 2023; Rotheneichner et al., 2018). By contrast, we report here a slow soma shrinkage, and AIS shortening, occurring over months and common to both AM and NM. These changes are unlikely due to maturation, and more likely due to aging. Age‐related shrinkage could well explain the larger soma of late AM, compared with AM, and the slightly longer AIS. Indeed, in reference to the time of awakening, late AM were about 6 months younger than the age‐matched AM (as measured at 15 months). Accordingly, the size of late AM soma was approx. 130% of that of AM, just as the size of younger NM (T1) was approx. 120% of that of older NM (T2). In addition, functional traits further underscored the relative immaturity of late AM, which had higher input resistance, smaller rheobase, and sparser synaptic input than AM. The same differences distinguish younger (less mature) and older (more mature) AM during the early adulthood, from 3 to 9 months of age (Benedetti et al., 2019). On the other hand, AM had an advantage of 6 months on late AM to refine their morphology and functional traits, and to age.

Our findings raise hope for an unsuspected plasticity in the old brain; however, they also lead to questioning about mechanisms allowing dormant precursors to shun maturation for most of a lifespan, and triggers of late awakening. According to what is known about adult neurogenesis, the awakening of precursors outside the neurogenic niches could be controlled by a plethora of intrinsic and extrinsic signals similar to those acting in the neurogenic niches (Abbott & Nigussie, 2020). However, environmental and/or behavioral triggers of maturation remain currently speculative and further studies concerning the possible modulation of the dormant precursor awakening are needed.

Nevertheless, studies of neuromodulation in the olfactory system hint toward what controls protracted immaturity and late awakening and help discuss the physiological meaning of AM. Indeed, previous research suggests that the awakening and maturation of AM are subject to extrinsic modulatory input, including different monoamines (Coviello et al., 2020, 2021; Gómez‐Climent et al., 2011; Vadodaria et al., 2017). In light of our data, such modulation must occur lifelong as neither awakening nor maturation are complete within the age of youth. At the same time, age‐related changes in neuromodulation, or receptor expression, must reflect the slowness of the awakening process. There are several forms of neuromodulation in the murine olfactory system subject to chronic oscillation. For instance, noradrenergic modulation participates in odor habituation during olfactory learning, while increased cholinergic neuromodulation partakes into age‐dependent scaling mechanisms that compensate for decline in odor quality (Brunert & Rothermel, 2021; Guerin et al., 2008; Mandairon et al., 2011). These processes would well fit to the slow and age‐related modulation of the AM awakening process. In relation to aging and late awakening, the existence of late AM implies that dormant precursors are relevant beyond the age of reproductive fitness, which in a mouse corresponds to ca. 2–8 months of age (Dutta & Sengupta, 2016). Is was proposed that maturing AM are essential to boost network plasticity and cope with variable environmental inputs (Bonfanti & Nacher, 2012; La Rosa & Bonfanti, 2021). Therefore, the protracted immaturity itself and the extremely slow AM maturation may serve to enhance brain plasticity over long periods, resulting in the delayed reach of maturity beyond the age of reproductive fitness.

Alternatively, the slow rate of awakening could be peculiar to mice reared in captivity and relative sensory deprivation. Under such assumption, we could speculate on the role of AM according to the period of most frequent awakening: the earlier part of life. Strikingly, most awakening events herald the reach of reproductive fitness, as observed in mouse (Rotheneichner et al., 2018) and as suggested by studies of human amygdala (Sorrells et al., 2019) and other mammalian brains (La Rosa et al., 2020). In the human amygdala, the loss of precursor immaturity during adolescence was proposed to aid plasticity critical for adult behavior and context‐dependent decision‐making (Keefer et al., 2021; Sorrells et al., 2019). In mice, abundant precursor between 2 and 4 months (Benedetti et al., 2019; Rotheneichner et al., 2018) heralds sexual maturity (Dutta & Sengupta, 2016). We therefore propose that in the mouse piriform cortex, the brain region explored in our work, AM serve for olfactory‐based association and learning, useful for exploration, foraging, socio‐sexual behavior, and predator avoidance (Kemble & Bolwahnn, 1997; Latham & Mason, 2004; Nevison et al., 2000). Assuming that the slow maturation and the very existence of late AM can be justified by the scarce brain stimulation in caged captivity, we predict that the faster maturation process and awakening rate will depend on environmental cues relevant for survival and reproductive fitness, which are recurrent in feral conditions.

In a third scenario, we consider dormant precursors as an emergency resource to cope with pathophysiology. In spite of many previous speculations, the only experimental attempt to control dormant precursor awakening through olfaction and behavioral (olfactory) conditioning so far failed altogether. By contrast, awakening of dormant precursors in the olfactory cortex was seemingly promoted by trauma, that is, transection of the olfactory bulb, which coincided with a waning of immature neuronal marker expression and with an increase in mature marker expression (Gómez‐Climent et al., 2011). The coincidence of awakening with trauma that reputedly causes loss of principal neurons in the olfactory cortex (Hedley & Brynmor, 1962) hints toward the role of dormant precursors as emergency resource to cope with accidental loss of neonatal‐matured neurons. In line with this speculation, slowly integrating precursors might have compensatory effects for functional losses, for example, during aging (Mandairon et al., 2011). Yet, such a pathophysiology‐related scenario is difficult to reconciliate with the uneven distribution of dormant precursors across the CNS and with their lack of proliferation (König et al., 2016), either of which properties result in inefficient regenerative potential. Furthermore, the extreme sparseness of residual late dormant precursor in the aged brain speaks against their relevance for age‐related pathophysiology, whereas similar limitations are also common to the regenerative pool of the neurogenic niches, subject to age‐dependent waning (Winner et al., 2011). On the contrary, we now know that, unlike rodents, in large‐brained mammals, including primates (La Rosa et al., 2020) and humans (Coviello et al., 2021; Li et al., 2023), dormant neurons are more abundantly distributed in the whole neocortex, and still maintained at adult/old stages (La Rosa et al., 2020; Li et al., 2023). In the light of results reported in the present study, these interspecies differences suggest that a pool of dormant neurons might awaken and integrate the brain of adult/old primates as well. Finally, some differences between AM and late AM may be attributed to a slow or incomplete maturation of late AM: if traits such as increased excitability and scarce synaptic input of late AM reflected incomplete maturation, rather than late awakening, slow maturation and scarce synaptic integration, may somehow limit the relevance of latecomer neurons for the function of pre‐existing networks after a certain age.

In conclusion, dormant precursor awakening and maturation occur lifelong and regardless of brain aging. Thus, dormant precursors are a versatile resource for adult and aged brain maturation and plasticity. However, the extreme delay of some awakening events and the slow AM maturation in the aging brain are puzzling, especially if we attempt to explain the role of these cells in terms of evolutionary advantage. Deciphering the behavioral and functional relevance of immature AM as well as controlling the rate of maturation will likely resolve some uncertainties and allow to harness this brain resource in relation to environment, behavior, and/or pathology.

## AUTHOR CONTRIBUTIONS

Bruno Benedetti contributed to the project design, management, data acquisition and analysis, manuscript preparation, and revision. Maximilian Reisinger contributed to the data acquisition and analysis, and manuscript revision. Marie Hochwartner and Gabriele Gabriele contributed to the data acquisition and analysis, and manuscript revision. Dominika Jakubecova and Ariane Benedetti contributed to the data acquisition. Luca Bonfanti and Sebastien Couillard‐Despres contributed to the project design and manuscript revision.

## FUNDING INFORMATION

This project was possible thanks to the generous support of the Paracelsus Medical University of Salzburg (PMU), in the frame of the PMU Research and Innovation Fund (PMU‐RIF) Seed Money for Novel Innovative Ideas and Preparatory Projects (SEED); project number: 2021‐SEED‐003‐Benedetti.

## CONFLICT OF INTEREST STATEMENT

The authors declare no conflict of interest.

## Supporting information


Data S1.

## Data Availability

The data that support the findings of this study are available from the corresponding author upon reasonable request.
