# Supplementary material for: The awakening of dormant neuronal precursors in the adult and aged brain
Source: Aging Cell. 2023 Aug 30;22(12):e13974. doi: 10.1111/acel.13974 (PMC10726842; doi:10.1111/acel.13974)
Supplement: Supplementary file 1 — Data S1. [file ACEL-22-e13974-s001.docx]

**Supporting Information**

**Supplementary Table 1:** analysis of dormant precursor morphology upon aging

|  | **T1** | | | **T2** | | | **T3** | | | **P values** |
| --- | --- | --- | --- | --- | --- | --- | --- | --- | --- | --- |
|  | Average | SD | N | Average | SD | N | Average | SD | N |  |
| **Cell density (cell/mm^3^)** | 11.8*10^3^ | 5.2*10^3^ | 40 | 10.4*10^3^ | 4.0*10^3^ | 40 | 13.5*10^3^ | 3.7*10^3^ | 40 | 0.6586^1^  0.0651^2^ |
| **Soma size (µm^2^)** | 153.2 | 49.35 | 1359 | 145.4 | 42.24 | 1179 | 125.2 | 33.64 | 504 | < 0.0001^1^  < 0.0001^2^ |
| **AIS length (µm)** | 26.7 | 6.0 | 52 | 20.6 | 10.0 | 26 | 17.9 | 7.3 | 46 | 0.0059^1^  < 0.0001^2^ |
| **Dendrite length (µm)** | 104.1 | 68.06 | 154 | 85.2 | 63.7 | 187 | 96.2 | 67.0 | 170 | 0.0101^1^  0.5978^2^ |
| **Dendritic spines (spine/µm)** | 0.8 | 0.2 | 30 | 0.7 | 0.2 | 30 | 0.8 | 0.2 | 30 | > 0.9999^1^  > 0.9999^2^ |
| **Syn/spine (%)** | 78.1 | 13.7 | 30 | 85.1 | 7.7 | 30 | 83.8 | 6.7 | 30 | 0.0323^1^  0.3693^2^ |
| ^1^Post-hoc test comparison of T1 and. T2; ^2^Post-hoc test comparison of T1 vs. T3 | | | | | | | | | | |

**Supplementary table 2:** analysis of NM morphology upon aging

|  | **T1** | | | **T2** | | | **T3** | | | **P values** |
| --- | --- | --- | --- | --- | --- | --- | --- | --- | --- | --- |
|  | Average | SD | N | Average | SD | N | Average | SD | N |  |
| **Soma size (µm^2^)** | 195.7 | 43.3 | 40 | 159.8 | 30.3 | 60 | 132.3 | 13.3 | 40 | 0.0008^1^  < 0.0001^2^ |
| **AIS length (µm)** | 29.3 | 7.1 | 766 | 22.7 | 5.8 | 784 | 20.6 | 6.1 | 1061 | <0.0001^1^  < 0.0001^2^ |
| ^1^Post-hoc test comparison of T1 and. T2; ^2^Post-hoc test comparison of T1 vs. T3 | | | | | | | | | | |

As observed for dormant precursors, age-related shrinkage of the soma and shortening of the AIS occur in NM as well. Moreover, the cell soma of NM was significantly larger than that of age-matched AM for any age group (P < 0.01). Conversely, no significant difference occurred comparing the AIS of NM and age-matched AM for any age group (P > 0.1).

| 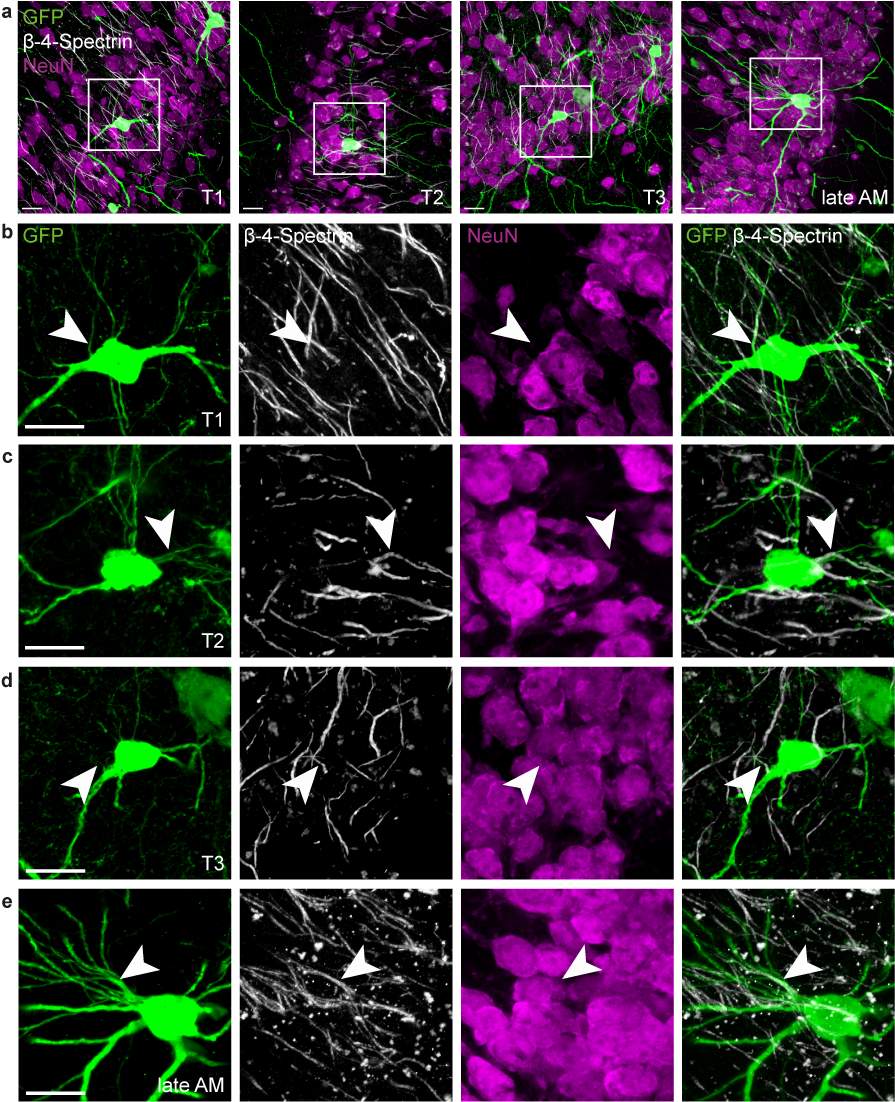 |
| --- |
| **Supplementary figure 1. Adult-mature neurons (AM) at different ages (T1, T2, T3, and Late AM) express mature neuron marker NeuN.** The scaffolding protein β4-spectrin outlines the axon initial segment of green neurites, pinpointing the position of the axon. A. matured dormant precursors are displayed at lower magnification EGFP signal is shown in green, β4-spectrin is shown in white and NeuN is shown in magenta. B-E details from the captions framed in white, in A, are displayed as single channels to outline finer details of soma and axon of AM |
